# Simultaneous colonic metastasis of advanced gastric cancer: a case report

**DOI:** 10.1186/s40792-023-01622-x

**Published:** 2023-03-17

**Authors:** Takafumi Shima, Asami Arita, Satoshi Sugimoto, Shoichi Takayama, Nao Kawaguchi, Yoshiro Imai, Tomohiro Kitahara, Tamaki Maeda, Junji Okuda

**Affiliations:** 1 Minimally Invasive and Robot Surgery Center, Toyonaka Keijinkai Hospital, 1-8-12 Shoji, Toyonaka, Osaka 560-0004 Japan; 2 Department of General and Gastroenterological Surgery, Osaka Medical and Pharmaceutical University, 2-7 Daigaku-machi, Takatsuki, Osaka 569-8686 Japan; 3 Department of Gastroenterology, Toyonaka Keijinkai Hospital, 1-8-12 Shoji, Toyonaka, Osaka 560-0004 Japan; 4 Department of Pathology, Toyonaka Keijinkai Hospital, 1-8-12 Shoji, Toyonaka, Osaka 560-0004 Japan

**Keywords:** Colonic metastasis, Gastric cancer, Poorly differentiated adenocarcinoma, Signet ring cell carcinoma

## Abstract

**Background:**

Although distant metastasis in gastric cancer can be present at the time of the initial diagnosis, colonic metastasis is extremely rare. This report describes a case of simultaneous colonic metastasis of advanced gastric cancer.

**Case presentation:**

The patient was a 78-year-old woman with nausea and epigastric pain. Upper gastrointestinal endoscopy revealed an advanced invasive ulcerative tumor in the lesser curvature of the stomach extending from the anterior to the middle portion. Colonoscopy revealed a 4-mm polyp-like lesion in the mid-transverse colon; therefore, a polypectomy was performed. Both gastric and colonic tumors showed poorly differentiated adenocarcinoma with signet ring cell carcinoma. After providing informed consent, the patient underwent a total gastrectomy. Histologic examination showed similar morphologic features of both gastric and colonic tumors. Immunohistochemistry staining showed that these tumor cells were positive for cytokeratin (CK) 7 and negative for CK20.

**Conclusions:**

This was an extremely rare case of simultaneous colonic metastasis of advanced gastric cancer. Because missed metastasis can result in a poorer prognosis, we propose a systemic search including colonoscopy for patients with advanced gastric cancer, especially cases involving poorly differentiated adenocarcinoma or signet ring cell carcinoma.

**Supplementary Information:**

The online version contains supplementary material available at 10.1186/s40792-023-01622-x.

## Background

Gastric cancer remains a critical disease that causes many deaths worldwide [1]. Distant metastases spreading to the liver (48%), peritoneum (32%), lung (15%), and bone (12%) are found in 30–35% of patients at the time of the initial clinical observation [2]. However, metastases to the colon are extremely rare [3, 4]. We report a case of simultaneous colonic metastasis of advanced gastric cancer.

## Case presentation

**Fig. 1:**
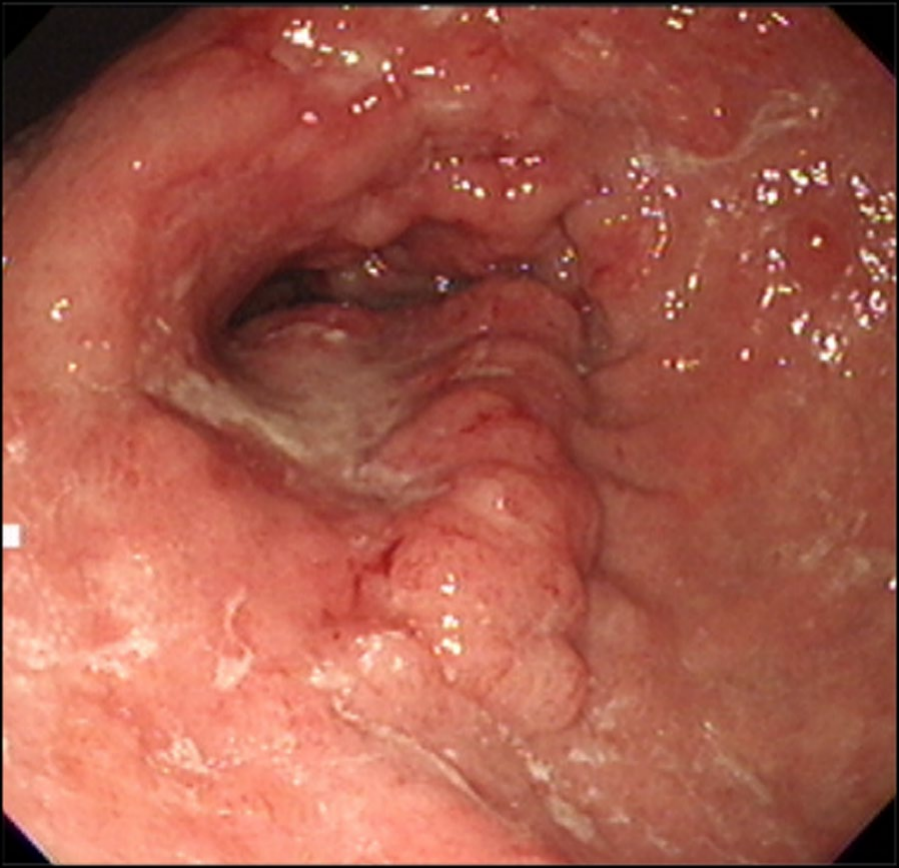
Upper gastrointestinal endoscopy revealed an advanced infiltrative ulcerative tumor in the lesser curvature extending from the antrum to the middle of the body of the stomach

**Fig. 2:**
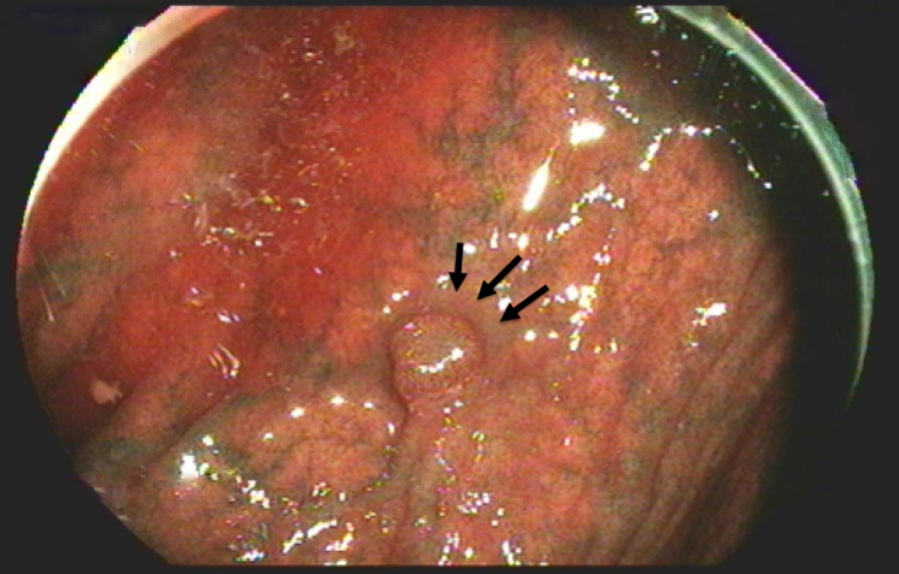
Colonoscopy revealed a 4-mm polyp in the mid-transverse colon (arrows)

**Fig. 3:**
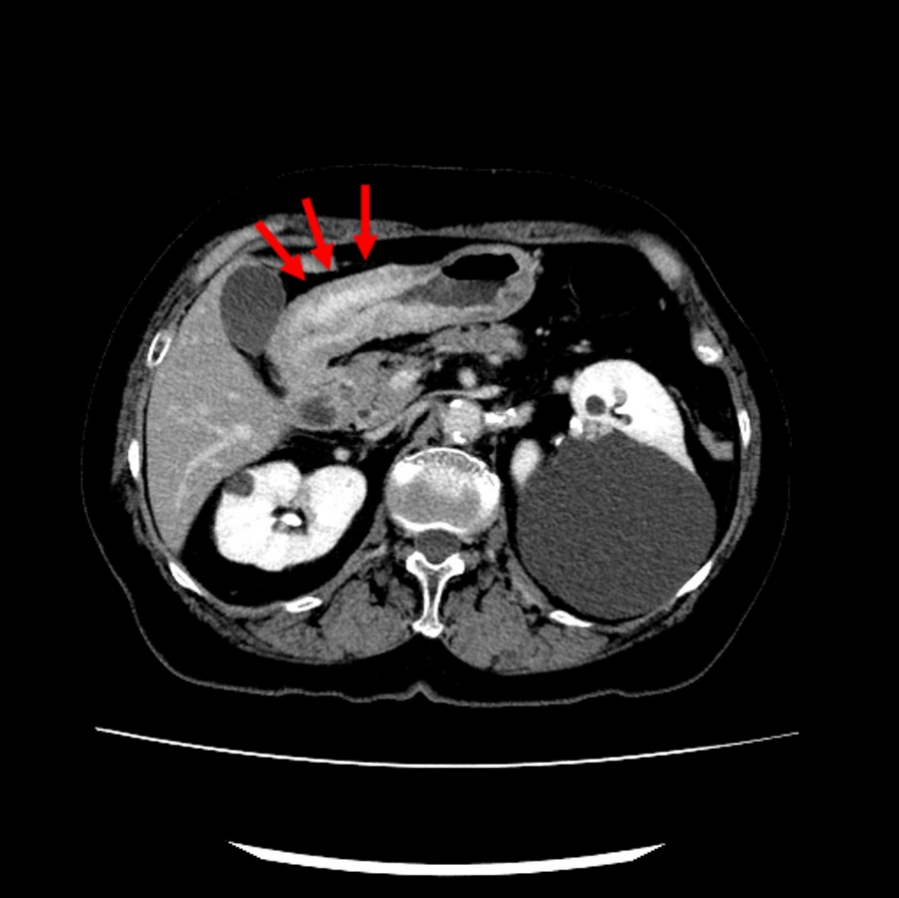
Computed tomography revealed enhanced wall thickening in the lesser curvature of the stomach from the antrum to the middle of the body (red arrows)

**Fig. 4:**
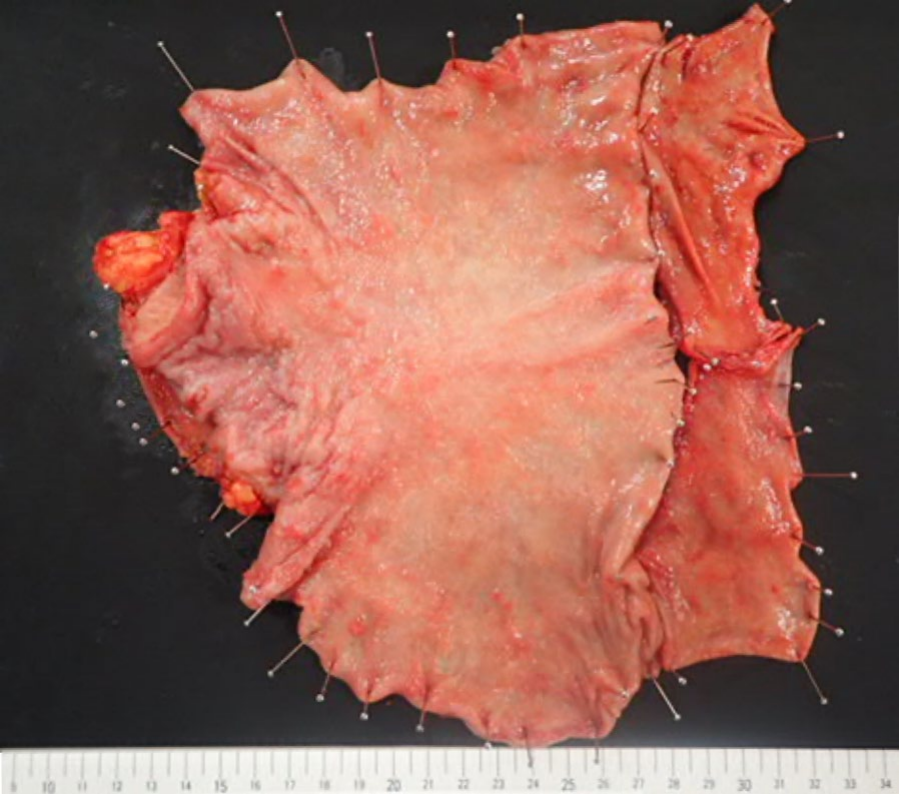
Surgical specimen revealed a type 3 gastric tumor centered in the antrum and pylorus measuring 180 mm × 170 mm

**Fig. 5:**
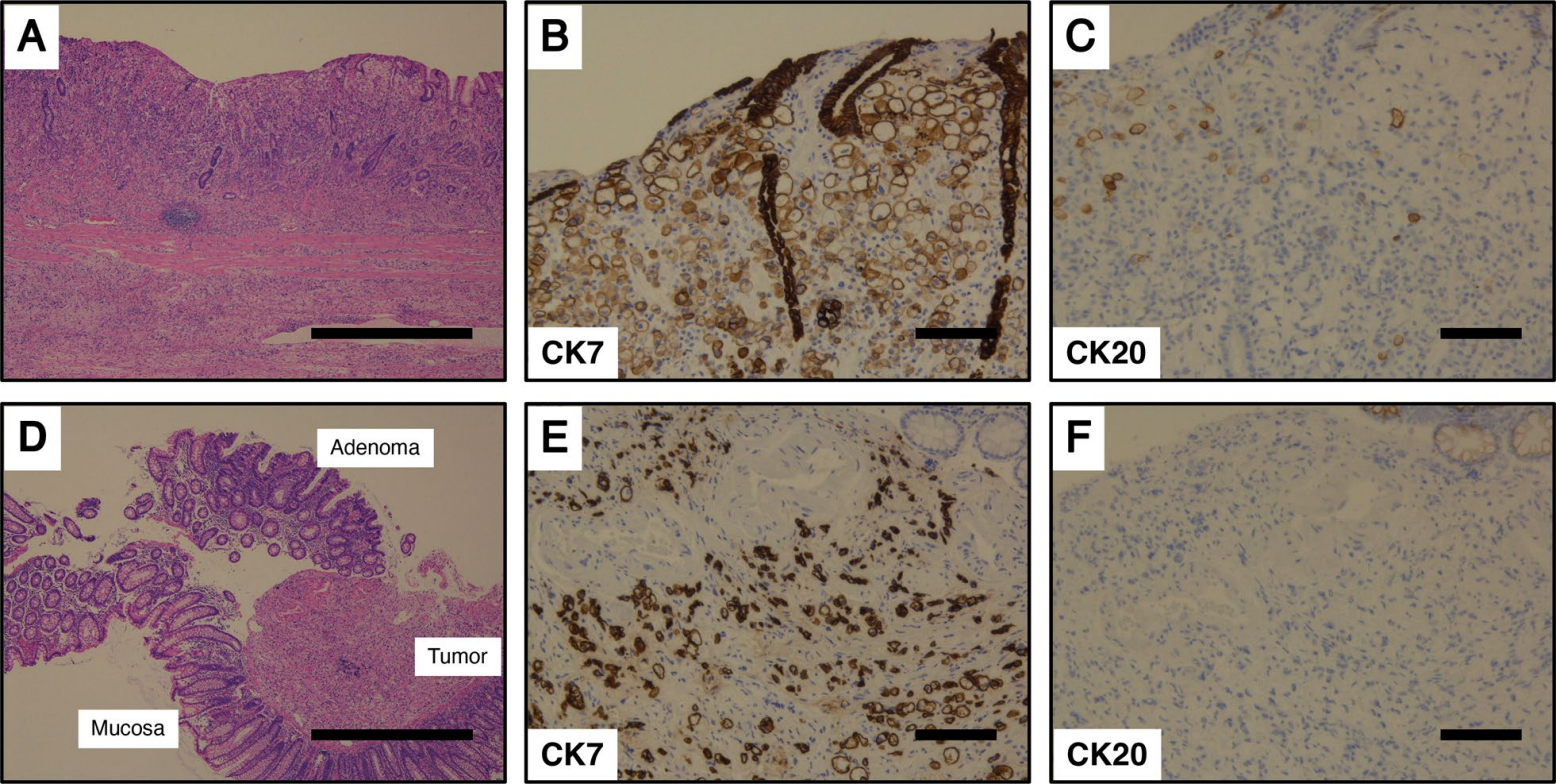
**A**–**F** Histologic examinations of the gastric and colonic tumors. **A** Hematoxylin and eosin staining of the gastric tumor shows poorly differentiated adenocarcinoma with signet ring cell carcinoma (×20). Scale bar, 1000 μm. **B**, **C** Immunohistochemistry staining of the gastric tumor cells yields positive results for CK7 (**B**; ×200) and negative results for CK20 (**C**; × 200). Scale bar, 100 μm. **D** Hematoxylin and eosin staining of the colonic tumor shows poorly differentiated adenocarcinoma with signet ring cell carcinoma (×20). The mucosal surface of the colonic tumor shows normal mucosa with partial features of adenoma. Scale bar, 1000 μm. **E**, **F** Immunohistochemistry staining of the colonic tumor cells yields positive results for CK7 (**E**; ×200) and negative results for CK20 (**F**; ×200). Scale bar, 100 μm. *CK* cytokeratin

The patient was a 78-year-old woman who presented to her primary care physician with nausea and upper abdominal pain. She was referred to our hospital for further examination and treatment. There was no notable family history. Laboratory data showed mild elevation of a single tumor marker (carcinoembryonic antigen, 1.9 ng/mL; carbohydrate antigen 19-9, 2.0 U/mL; carbohydrate antigen 125, 44.5 U/mL; alpha-fetoprotein, 8.9 ng/mL) and no other significant abnormal findings. Upper gastrointestinal endoscopy showed an advanced infiltrative ulcerative tumor in the lesser curvature extending from the antrum to the middle of the body of the stomach (Fig. 1). Endoscopic biopsy of the gastric tumor showed poorly differentiated adenocarcinoma with signet ring cell carcinoma. Colonoscopy showed a 4-mm polyp in the mid-transverse colon (Fig. 2). Cold snare polypectomy further revealed poorly differentiated adenocarcinoma with signet ring cell carcinoma. Computed tomography displayed enhanced wall thickening in the lesser curvature of the stomach from the antrum to the middle of the body (Fig. 3). The regional lymph nodes showed no enlargement. Magnetic resonance cholangiopancreatography showed no obvious abnormal findings in the gallbladder, cystic duct, or common bile duct (Additional file 1). We considered this to be a case of colonic metastasis of gastric cancer. Although further examinations using fluorine-18 fluorodeoxyglucose positron emission tomography and computed tomography were not performed, the patient had a gastric obstruction that was considered to be advanced gastric cancer. Because it was feared that bleeding from the tumor would occur, a gastrectomy was planned. Intraoperatively, the diagnostic peritoneal washing cytology examination yielded negative results for malignant cells, and no peritoneal dissemination was observed. We attempted a distal gastrectomy; however, a positive intraoperative rapid pathology diagnosis led to a total gastrectomy, lymph node dissection, and cholecystectomy. Gastric cancer did not directly invade the transverse colon. Because the location of the colonic lesion was difficult to determine, colon resection was not performed. The surgical specimen revealed a type 3 gastric tumor centered in the antrum and pylorus that was 180 mm × 170 mm, with no grossly apparent abnormalities in the gallbladder (Fig. 4, Additional file 2). The histologic examination showed similar morphologic features of the gastric and colonic tumors (Fig. 5). Hematoxylin and eosin staining revealed poorly differentiated adenocarcinoma with signet ring cell carcinoma. The mucosal surface of the colonic tumor was covered by normal mucosa with partial features of adenoma. In addition, immunohistochemistry showed that these tumor cells were positive for cytokeratin (CK) 7 and negative for CK20. In the gastric tumor, human epidermal growth factor receptor 2 was negative, the combined positive score of programmed death ligand-1 was 1–5, and high microsatellite instability was not observed (data not shown). In addition to advanced lymph node metastasis and lymphatic and venous invasion, tumor invasion was seen at the proximal and distal margins or within the wall of the gallbladder neck (microscopic residual tumor). According to the Japanese classification of gastric carcinoma, the final pathological diagnosis was poorly differentiated adenocarcinoma with signet ring cell carcinoma, pT4a, INFc, Ly1a, V1a, pPM1, pDM1, and pN3a (3: 4/4; 5: 1/1; 6: 4/4; 12a: 1/1; total: 10/31) [5].

The patient was discharged from the hospital on postoperative day 21 without adverse events. Chemotherapy comprising 5-fluorouracil, leucovorin, and oxaliplatin plus nivolumab was initiated.

## Conclusions and discussion

Colonic metastases with gastric cancer as the main etiology are extremely rare, as are colonic metastases with breast, renal, prostate, and ovarian cancers as the main etiology [4, 6]. We performed a literature search of colonic metastasis of gastric cancer cases using the PubMed database from January 1981 to November 2022. We used the following keywords and their combinations: “colonic metastasis”, “colon metastasis”, “poorly differentiated”, “signet ring cell”, and “gastric”. The search revealed only ten reports, suggesting that colonic metastasis of gastric cancer is very rare or often misdiagnosed [3, 7–15]. One report summarized 21 cases of colorectal recurrence more than 5 years after the resection of gastric cancer, whereas another report summarized 14 cases of colonic metastases of gastric cancer [3, 8]. The metastases sites varied from the cecum to the rectum, and the number of metastases varied from single to multiple. In addition, six cases were reported by five case reports [9, 10, 12, 14, 16]. These reports indicated that the majority of gastric cancers that developed colonic metastases were poorly differentiated adenocarcinoma or signet ring cell carcinoma; however, a relationship with alpha-fetoprotein-producing gastric cancer was not described. For our case, the pathological diagnosis was poorly differentiated adenocarcinoma with signet ring cell carcinoma with no apparent distant metastasis other than the transverse colon.

Major metastatic pathways from gastric cancer to the colon include the hematogenous, lymphogenous, and implantation pathways, specifically in the large intestine, from the gastrointestinal lumen. Implantation is the most common pathway [3]. In several cases, metastatic patterns of the gastrointestinal lumen showed multiple lesions, and these metastases often had a polypoid form [3, 4, 17]. In the present case, a single polyp-like colonic metastasis with a generally normal mucosal surface was noted. Because we did not perform additional surgery of the colon, it was difficult to determine the exact type of metastasis. However, based on the previous findings, we did not consider it to be a metastatic pattern of implantation in the large intestine through the gastrointestinal lumen.

Although signet ring cell carcinoma of the colon is rare, it is crucial to differentiate between primary colon cancer and colonic metastasis of gastric cancer. Tumor cells with a CK7(−)/CK20(+) immunohistochemical staining pattern indicate primary colon cancer, whereas a CK7(+)/CK20(−) staining pattern indicates primary gastric cancer [18]. Therefore, based on the histological features and immunohistochemical analysis results, colonic metastasis of gastric cancer was diagnosed.

The theoretical basis for preoperative colonoscopic examinations of patients with gastric cancer remains poor, with no established recommendations [19, 20]. Furthermore, when a colonoscopy of a patient with gastric cancer reveals a colonic lesion, it may be difficult to preoperatively determine whether the lesion is benign or malignant and whether it is primary or metastatic. In this case, primary gastric cancer was advanced, and it was difficult to achieve negative microscopic resection margins. However, complete resection of primary gastric cancer with colonic metastasis has been reported [3]. The necessity for additional resection is controversial; however, we believe that delayed detection of colonic metastasis of gastric cancer may lead to a poorer prognosis. Therefore, preoperative colonoscopy for possible colonic metastasis may be useful for patients with gastric cancer. Although small intestine metastasis of gastric cancer is extremely rare, it has been reported [21, 22]. Therefore, we believe that it is necessary to consider the invasiveness of the examination and medical costs when deciding whether to actively perform a detailed examination of the small intestine of patients with advanced gastric cancer.

In summary, we encountered an extremely rare case of simultaneous colonic metastasis of advanced gastric cancer. We propose a systemic search including colonoscopy for patients with advanced gastric cancer, especially cases involving poorly differentiated adenocarcinoma or signet ring cell carcinoma histologic types.

## Supplementary Information

**Additional file 1.** Magnetic resonance cholangiopancreatography shows no obvious abnormal findings in the gallbladder, cystic duct, and common bile duct.

**Additional file 2.** Surgical specimen showing no grossly apparent abnormalities in the gallbladder.

## Data Availability

Data sharing is not applicable to this article because no datasets were generated or analyzed during the current study.

## Abbreviation

CK: Cytokeratin
