# Supplementary figures and images for: Simultaneous colonic metastasis of advanced gastric cancer: a case report
Source: Surg Case Rep. 2023 Mar 17;9:39. doi: 10.1186/s40792-023-01622-x (PMC10023818; doi:10.1186/s40792-023-01622-x)

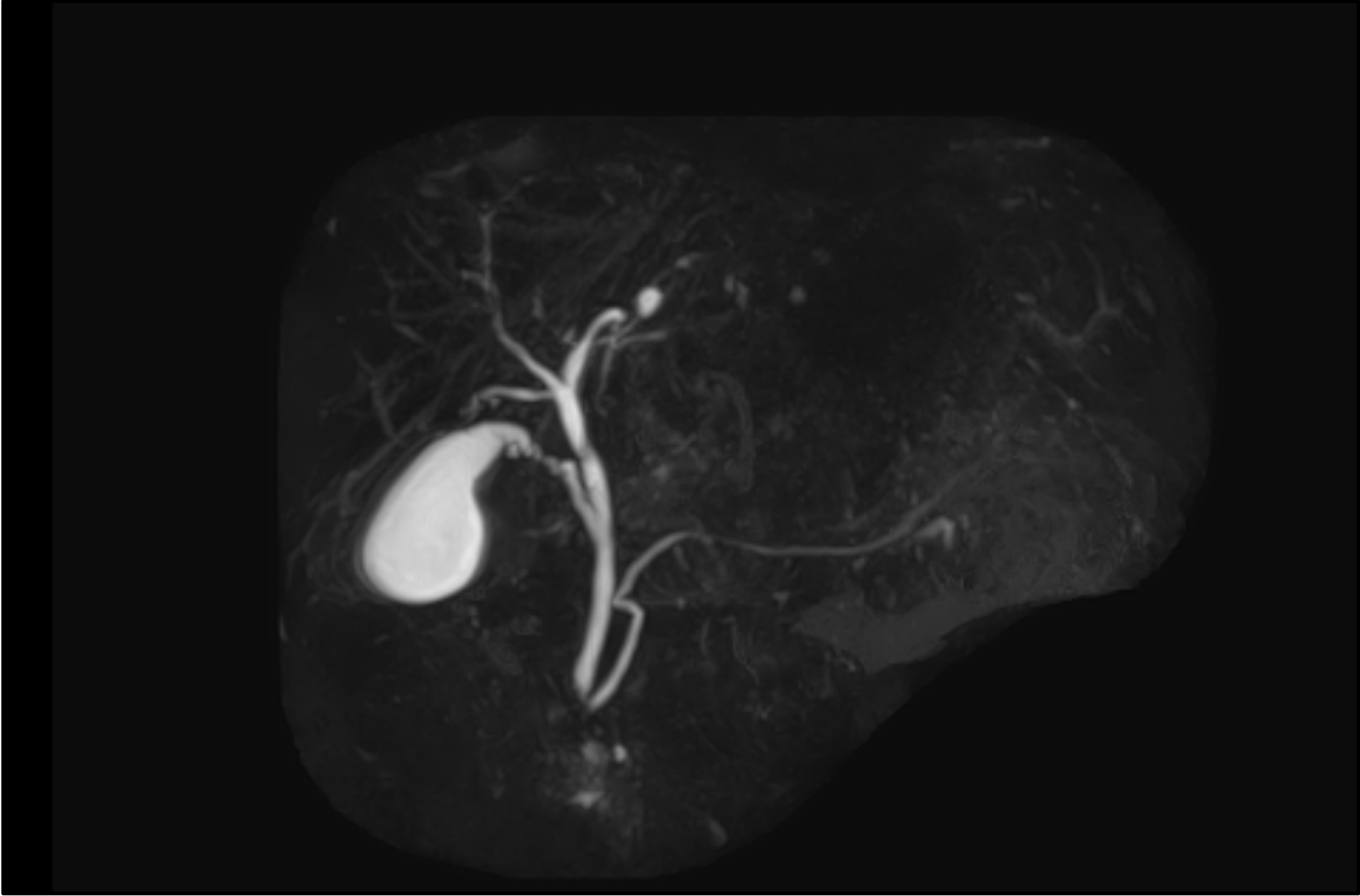

Supplement: Supplementary file 1 — Additional file 1. Magnetic resonance cholangiopancreatography shows no obvious abnormal findings in the gallbladder, cystic duct, and common bile duct. [file 40792_2023_1622_MOESM1_ESM.pdf]

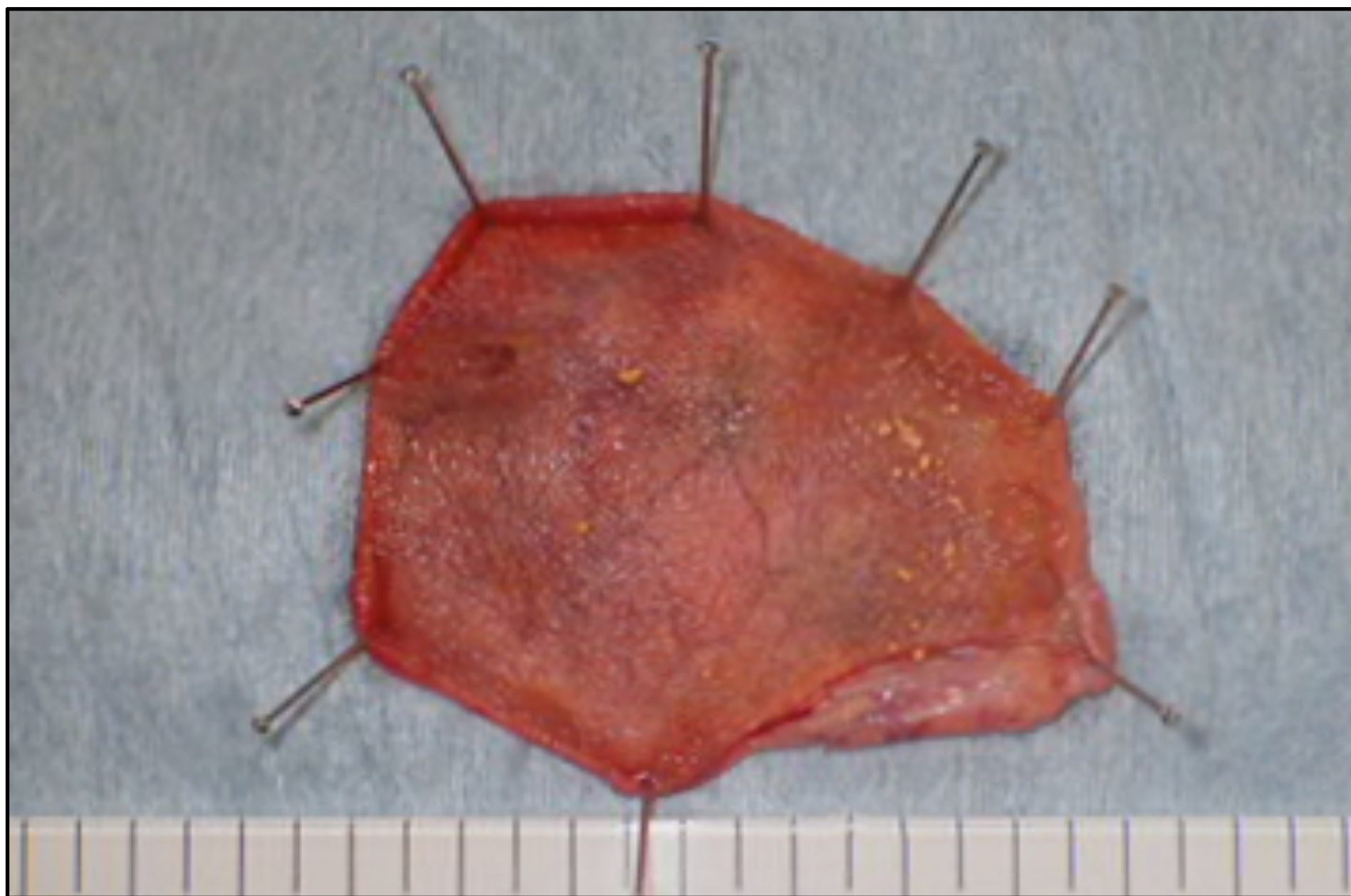

Supplement: Supplementary file 2 — Additional file 2. Surgical specimen showing no grossly apparent abnormalities in the gallbladder. [file 40792_2023_1622_MOESM2_ESM.pdf]
